# Carbon Fiber Reinforced Carbon–Al–Cu Composite for Friction Material

**DOI:** 10.3390/ma11040538

**Published:** 2018-03-31

**Authors:** Lihui Cui, Ruiying Luo, Denghao Ma

**Affiliations:** School of Physics and Nuclear Energy Engineering, Beihang University, Beijing 100191, China; ryluo@buaa.edu.cn (R.L.); denghaoma@163.com (D.M.)

**Keywords:** microstructure, flexural properties, electrical properties, friction coefficient, aluminum–copper alloy

## Abstract

A carbon/carbon–Al–Cu composite reinforced with carbon fiber 2.5D-polyacrylonitrile-based preforms was fabricated using the pressureless infiltration technique. The Al–Cu alloy liquids were successfully infiltrated into the C/C composites at high temperature and under vacuum. The mechanical and metallographic properties, scanning electron microscopy (SEM), X-ray diffraction (XRD), and energy dispersive spectroscopy (EDS) of the C/C–Al–Cu composites were analyzed. The results showed that the bending property of the C/C–Al–Cu composites was 189 MPa, whereas that of the pure carbon slide material was only 85 MPa. The compressive strength of C/C–Al–Cu was 213 MPa, whereas that of the pure carbon slide material was only 102 MPa. The resistivity of C/C–Al–Cu was only 1.94 μΩm, which was lower than that of the pure carbon slide material (29.5 μΩm). This finding can be attributed to the “network conduction” structure. Excellent wettability was observed between Al and the carbon matrix at high temperature due to the existence of Al_4_C_3_. The friction coefficients of the C/C, C/C–Al–Cu, and pure carbon slide composites were 0.152, 0.175, and 0.121, respectively. The wear rate of the C/C–Al–Cu composites reached a minimum value of 2.56 × 10^−7^ mm^3^/Nm. The C/C–Al–Cu composite can be appropriately used as railway current collectors for locomotives.

## 1. Introduction

Pure carbon slides (CY280, Morgan Advanced Materials, Co., Ltd., Shanghai, China) are the main slide used in electrified railways in China due to its excellent self-lubricating and wear-resisting properties, high-temperature resistance, and low density [1,2]. The flexural strength of a pure carbon slide ranges from 30 MPa to 90 MPa, and its compressive strength varies between 60 and 100 MPa due to relatively low mechanical properties. Therefore, the carbon slide is easily fractured and broken. In addition, the large inherent resistance, low collector capacity, and high temperature at the contact area of the pure carbon slide easily induces wire overheating oxidation corrosion, which can accelerate the wear speed and shorten the service life of the copper wire. In contrast, carbon/carbon composites are characterized by low density, high specific strength, high heat transfer conductivity, wear resistance, and high-temperature resistance [3]. These materials are widely used in aviation, aerospace, nuclear energy, chemistry, biomaterial, and other fields. To improve the advantages of C/C composites, numerous studies have been successively conducted on topics such as C/C composites and Cu alloys in the field of welding, C/C composite coating treatment, and the addition of refractory metal carbide in the matrix. Other studies on the infiltration of silicon into carbon/carbon composites to improve C/C oxidation resistance, ablation resistance, thermal shock resistance, and other properties have been conducted to expand the application of C/C materials [4,5,6]. For instance, copper–carbon composites are widely used as contact strips for pantographs, collector shoes in electric railways, and brushes for motor technology [7].

Several researchers have achieved the penetration of copper into graphite through pressurized infiltration to prepare a laryngeal lining material to improve the thermal shock resistance and ablation performance. C/C composites possess a large number of pores that can be used to infiltrate the copper alloy. Taking advantage of the high-temperature sweat property of copper alloy cannot only improve the ablation resistance, but also enhance the thermal shock resistance of C/C composites. Furthermore, C/C composites and copper are highly conductive and can be used in conductive and friction materials. However, the carbon matrix exhibits poorer wettability toward copper than aluminum. At high temperature, aluminum reacts with pyrolytic carbon to form Al_4_C_3_, which improves the wettability of the alloy liquid and the permeability of the molten alloy [8,9].

Other researchers have concentrated on pure graphite. Pure graphite is an excellent solid lubricant, and the use of graphite effectively decreases the friction coefficient. Moreover, graphite possesses a laminar structure, and a continuous film forms on the tribo-surface during sliding, thus preventing direct contact between two sliding surfaces, leading to a decreased wear rate [10,11,12,13,14,15,16,17]. However, pure graphite exhibits low strength and poor thermal shock resistance; therefore, the material cannot be appropriately used in railway current collectors.

In the present work, a carbon fiber-reinforced C/C–Al–Cu composite with improved mechanical properties and electrical conductivity was prepared through the pressureless infiltration technique (PLI). Then, the electrical and mechanical properties of the composite were measured. Finally, the excellent electrical conductivity and bending property of the composites were discussed in detail.

## 2. Experimental Procedure

### 2.1. Preparation of the Materials

**Figure 1:**
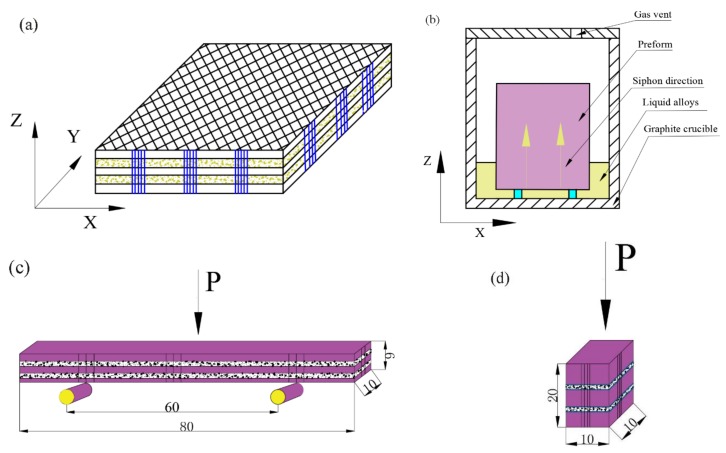
Schematic of the preform, siphon tooling mold, and test diagrams. (**a**) Schematic of 2.5D-polyacrylonitrile-based carbon fiber felts; (**b**) schematic view of the siphon tooling mold; (**c**) three-point bending diagram; and (**d**) the compression diagram.

2.5D-polyacrylonitrile-based carbon fiber felts were used as preforms with a density of approximately 0.55 g/cm^3^. Polyacrylonitrile carbon fiber (12K PANCF) was provided by the Tuozhan Company, Yantai, China. In the basic structure of the preform, the weft and the mesh were alternately laminated. The function of the needle is to pierce the fiber in the felt into the weftless cloth layer (Figure 1a). First, the preforms were densified using natural gas (mainly containing CH_4_) and C_3_H_8_ + N_2_ through chemical vapor infiltration (CVI) at 1080 °C to 1100 °C under a total pressure of 1–5 kPa. Second, the preform was densified by CVI until a density of 1.2–1.5 g/cm^3^ was achieved. Finally, the carbon fiber preform was heat-treated at 2300 °C for 2 h. The aluminum-based penetrant immersed in the C/C materials was mixed: 90 wt % Al (50–80 μm, ≥99.5 wt % purity), 10 wt % Cu (50–80 μm, ≥99.5 wt % purity). All powders were of reagent grade and milled for 2–3 h.

As described in Figure 1b, the preform was placed in a graphite crucible, and a certain amount of infiltration alloy was added. Then, the mixture was heat-treated at 1200 °C for 20 min under vacuum. At a high temperature, the liquid alloy was slowly immersed in the preform by capillary force, then the C/C–Al–Cu material with a density of 2.24 g/cm^3^ was prepared. Figure 1c shows the test diagram of the three-point bending sample. The span of the bending test was 60 mm, and *P* is the maximum load (N). Pure carbon slide material (CY280) was used as the reference material for the comparative experiment. The microstructure of the samples was analyzed by metallographic (OLYMPUS PMG3, Olympus Corporation, Tokyo, Japan), scanning electron microscopy (SEM S-4800, Hitachi, Tokyo, Japan), and energy dispersive spectroscopy (EDS). X-ray diffraction (XRD, Model D/Max 2500P, Rigaku, Japan) with Cu Kα radiation was used to analyze the phase structures.

### 2.2. Testing Procedures

The final density of the specimens was determined by the Archimedes technique first, the dried samples were weighed as *m*_1_ (g). Second, the samples were put in boiling water for 2 h, taken out and cooled, then weighted again as *m_a_* (g). Third, the samples were weighed in deionized water as *m_b_* (g). Finally, the density (ρ in g/cm^3^) of the samples was calculated by the following equation:

(1) $$\rho =\frac{m_{1}}{m_{a}-m_{b}}$$

The flexural properties of the composites were measured at ambient temperature by a three-point bending test with a span of 60 mm and loading speed of 0.5 mm/min. The specimens were formed as rectangular bars with dimensions of 80 × 10 × 6 mm [18]. The flexural strength was calculated according to the equation:

(2) $$\sigma_{z}=\frac{3FL}{2bh^{2}}$$

where $\sigma_{z}$ is the flexural strength (MPa); *L* is the span of the bend test (mm); *F* is the maximum load (N); and *b* and *h* are the width and the thickness of the specimen (mm), respectively.

The compressive test specimens were processed into 10 × 10 × 20 mm along the axial direction as shown in Figure 1d. Five tests were typically conducted for each type of sample to ensure reproducibility. The nominal compressive stress $\sigma_{t}$ was calculated from the following equations:

(3) $$\sigma_{t}=\frac{F}{S}\;\;$$

where $\sigma_{t}$ is the compressive strength (MPa); *F* is the maximum load (N); and *S* is the cross-sectional area (mm^2^).

The friction coefficient and wear rate were performed using a block-on-ring wear test machine (MM-200, Spai Tech, Jinan, China) [19]. The samples were in the form of cubic blocks sized 10 × 10 × 10 mm. Care was taken to ensure good surface finishing and that the two disc faces were parallel [7,20]. These blocks rubbed against a copper disc with an outer diameter of 20 cm and a thickness of 6 mm. Wear tests were carried out under dry sliding condition with a normal load of 70 N [21]. The speed of rotation was 200 rpm. Each specimen was weighed before and after every wear test on an electronic balance with 0.1 mg precision and then the mass loss was obtained. The wear rate was calculated with the wear mass loss divided by the time of the wear tests [10,22]. The frictional torque during the test was recorded. The cure of the friction coefficient was obtained by processing the data of the friction signal transformed via an A/D converter. The value of the friction coefficient was calculated using the following equation:

(4) $$\mu =\frac{M}{FR}$$

where μ is the friction coefficient; *M* is the friction torque (kg·cm); *F* is the applied load (kg); and *R* is the radius of steel ring (cm). The morphology of the worn surfaces was analyzed using a scanning electron microscope (SEM S-4800).

### 2.3. Electrical Resistivity Measurements

The electrical resistivity of the specimens was measured using a direct current and low electrical resistor (TH2512B, Tonghui, Shenzhen, China) according to the JB/T 2664.1-1999 standard [19,23]. The tested samples were processed into 50 × 5 × 2 mm^3^. In order to insure reproducibility, each specimen was measured five times to obtain the averaged value. The value of electrical resistivity was calculated using the following formula:

(5) $$\rho =\frac{RS}{L}$$

where ρ is the electrical resistivity (Ω·m); *R* is the electrical resistance (Ω) shown in the resistor; *S* is the area of the cross-section (m^2^); and *L* is the length of specimens (m).

## 3. Results and Discussion

### 3.1. Microstructure and Morphology

Carbon/carbon composites contain abundant pores or macrocracks, and the porosity of these composites generally reach 10–15%. From Figure 1a,b, the X-Y plane of the C/C composite was parallel to the surface of the liquid alloy direction. Figure 1c,d schematically illustrate the process of mechanical properties testing.

**Figure 2:**
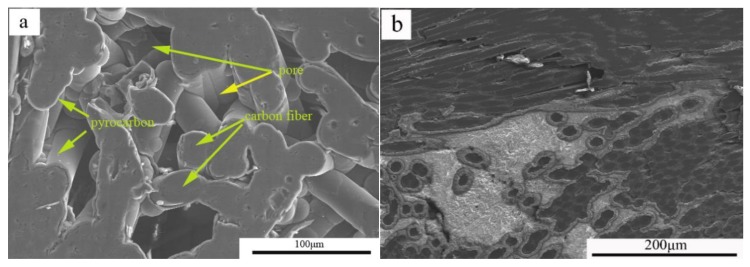

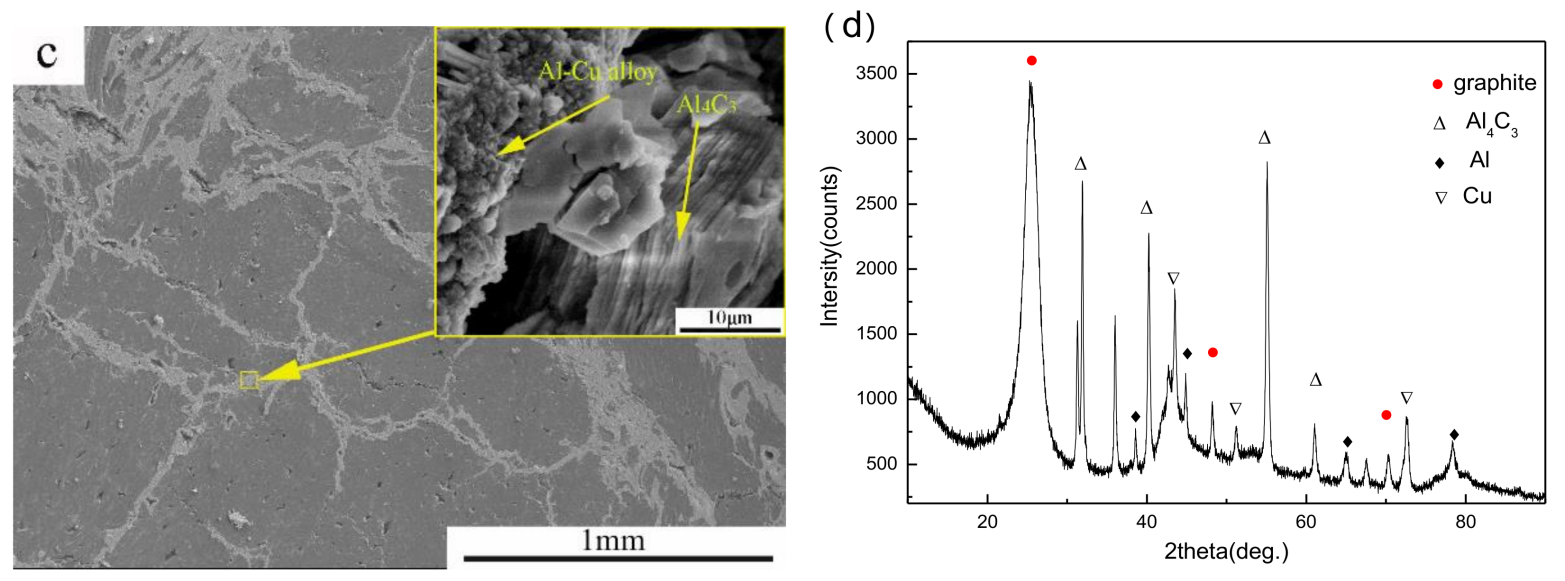
SEM micrographs of C/C, C/C–Al–Cu and XRD patterns: (**a**) Initial state of C/C; (**b**) metal infiltration of C/C; (**c**) formed metal network of C/C; and (**d**) XRD pattern of C/C–Al–Cu.

Figure 2a shows the initial state of the C/C composite samples, and Figure 2b shows that molten metal penetrated into the C/C composites. The molten metal of the composite formed a “network conduction” as shown in Figure 2c,d shows the XRD pattern of the proposed composite. The results showed that four phases existed: C, Al, Al_4_C_3_, and Cu.

The interface of the pyrolytic carbon and metal layers was compacted, and the “network conduction” structure was formed in the carbon matrix. The alloy liquid filled the interstices of the C/C composite by capillary force at high temperature and under vacuum. The continuous and uniform electrical network of the aluminum–copper phase formed in the interstices. 

### 3.2. Mechanical Properties and Fracture Behavior of the Composites

**Figure 3:**
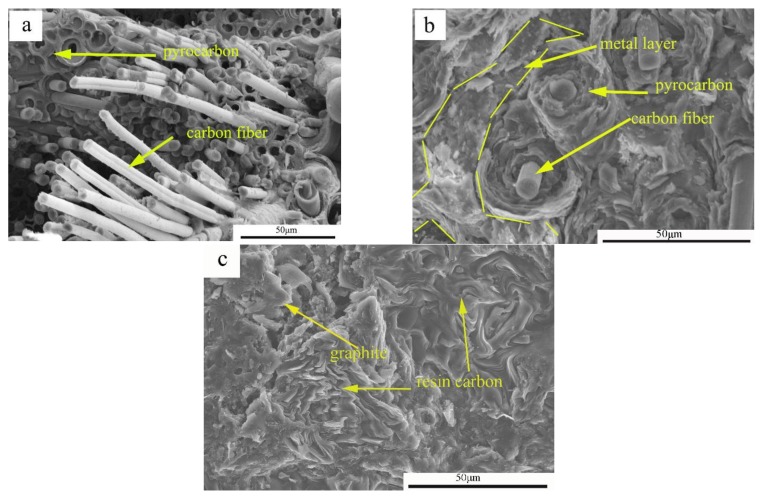
Fracture micro-morphology of C/C, C/C–Al–Cu, and pure carbon slide composites. (**a**) Bending fracture morphology of C/C; (**b**) bending fracture morphology of C/C–Al–Cu; and (**c**) bending fracture morphology of pure carbon slide.

As shown in Figure 3a, a large number of fiber pull-outs were observed on the fracture surface of the C/C composites. The fracture length of the fracture fiber was at the millimeter level because the interfacial bonding strength was relatively weak; thus, the composite material did not maximize the toughening effect during the fracture process.

Compared to the bending fracture of the C/C composite material, C/C–Al–Cu materials with fiber lengths at the micron level showed that carbon fiber debonding left cracks or holes as shown in Figure 3b. In the extraction of the carbon fiber surface, the pyrolytic carbon and aluminum–copper alloy were visible, indicating that the aluminum infiltration enhanced the bonding strength between the pyrocarbon and Al–Cu alloy. As the interfacial bonding strength of the aluminum and carbon was restricted, the carbon fiber in the process of viscous resistance was limited, resulting in the pullout of short carbon fibers [18]. The difference in interfacial bonding strength between C/C and C/C–Al–Cu was due to the excellent wettability of aluminum and the improved strengthening effect of the matrix, thereby resulting in enhanced fracture stress. However, as shown in Table 2, the flexural strength of the pure carbon slider was only 85 MPa, whereas that of C/C–Al–Cu was 189 MPa (loaded in the *Z*-direction). From Figure 3c, the fracture surface exhibited brittle fracture behavior, and only a small quantity of the graphite particles of the matrix was displayed. The results exhibited the poor bending property of the pure carbon slide. Compared with industry standards (Table 1), the flexural strength of the pure carbon pantograph exceeded 80 MPa, whereas the C/C–Al–Cu composite satisfied the requirements for pantograph sliders [19,24,25,26]. In Table 2, the compressive strength of C/C–Al–Cu was 213 MPa, whereas that of the pure carbon slide material was only 102 MPa. Finally, C/C–Al–Cu exhibited better mechanical properties than the pure carbon slide. 

The fracture surface of C/C is shown in Figure 3a. According to the theory of composite materials, the fracture mechanism of the C/C was the increase in test load along with displacement. When the maximum load was reached, the matrix was broken, and interfacial damage occurred constantly. The initial crack was deflected, and cracks propagated along the fiber direction, thereby causing fiber pullout or breakage. The C/C fracture surface, which was characterized by a large number of single-fiber pullouts, indicated weak interfacial bonding strength between the carbon fibers and pyrolytic carbon [27,28].

Excellent interfacial bonding strength is essential for the pyrolytic carbon/Al interface debonding and sliding, which can reduce stress concentration [29]. In the present work, excellent wettability between the pyrolytic carbon and aluminum–copper alloy was observed. Interfacial bonding strength was improved by the added Al, which formed the Al_4_C_3_ compound on the surface of the pyrolytic carbon. Normally, interfacial bonding involves mechanical bonding due to the poor wettability between pyrolytic carbon and the Cu alloy. The formation of carbide increased the bonding strength between the pyro-carbon and the Al–Cu alloy. In general, the interfacial reaction products, including elemental C, exert adverse effects on the mechanical properties of the composites by damaging the structure of the carbon fibers and causing increased interfacial bonding [23,30]. From Figure 3b, the fracture surface of the C/C–Al–Cu composites with carbides at the interface typically presented brittle characteristics. The pure carbon slider (CY280) exhibited the brittle behavior shown in Figure 3c.

**Figure 4:**
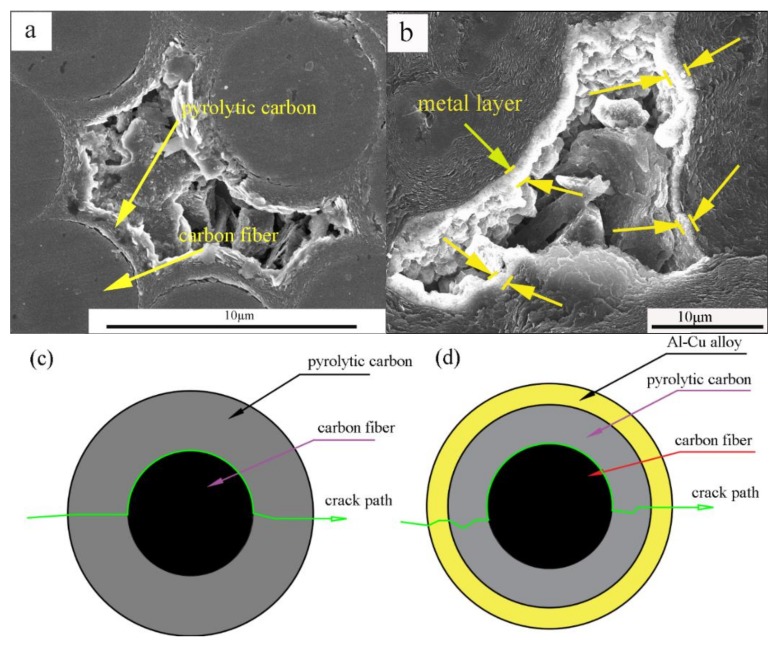
SEM micrographs of composite materials and Fracture mechanism model. (**a**) The microstructure morphology of C/C; (**b**) the microstructure morphology of C/C–Al–Cu; (**c**) the fracture mechanism diagram of C/C; and (**d**) the fracture mechanism diagram of C/C–Al–Cu.

**Figure 5:**
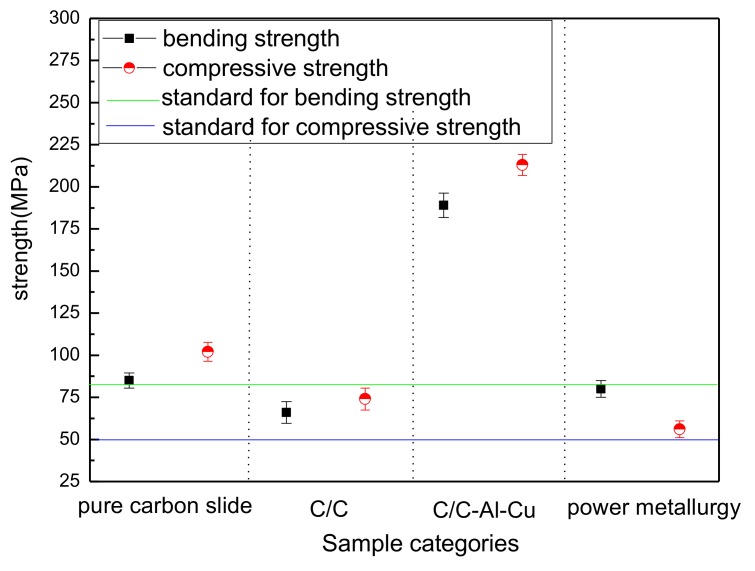
Testing error of the mechanical performance of samples.

Figure 4a,c and the schematic show the fracture process. Cracking appeared only through the carbon matrix, carbon fiber, and the interface bonding layer. As shown in Figure 4b,d, a layer of metal was uniformly wrapped around the pyrolytic carbon. First, the initial crack defects were decreased. Second, the increase in the path of crack propagation led to increased energy consumption during the process [3]. Finally, the carbon fiber facilitated strength and toughness. Figure 5 shows the mechanical performance of the different samples. The results demonstrated small errors in the experimental data and high repeatability. The industry standard for pantograph materials are shown in Table 1 [19,24,25,26].

### 3.3. Friction Performance and Frictional Behavior of the Composites

**Figure 6:**
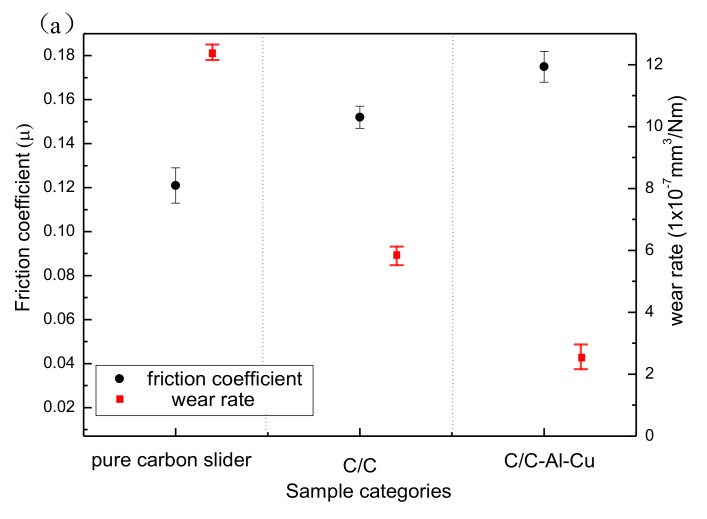

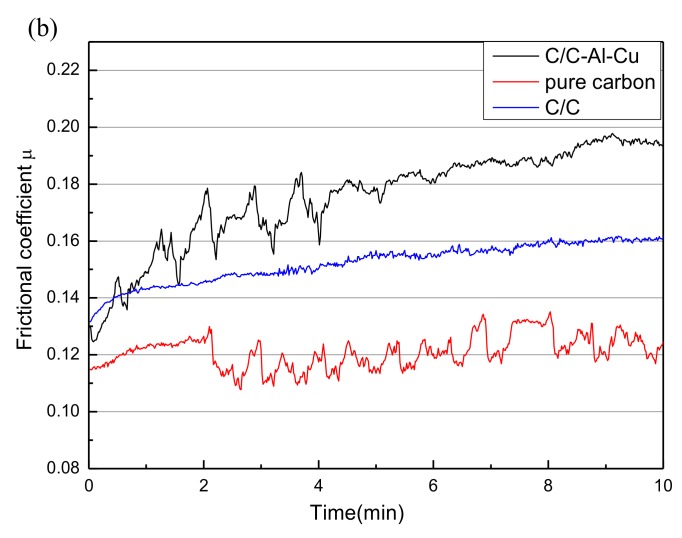
Friction coefficient for different samples. (**a**) Friction coefficient and wear rate; and (**b**) friction coefficient curve.

Figure 6a shows the variation in friction coefficient and wear rate of the composites. The friction coefficient of the composites increased monotonically. The pure carbon slide composite was mainly composed of carbon, graphite, and pitch coke. Graphite is an excellent solid lubricant; therefore, the addition of graphite effectively decreased the friction coefficient of the composites.

Figure 6b shows three kinds of friction curves under the load of 70 N and lubricated under dry conditions. The test time was controlled at 10 min. A duration of 3 min was required for the C/C composite to reach a stable state; this time was shorter than the required 6 min for the C/C–Al–Cu composite. For the pure carbon slide, the friction coefficient always changed irregularly, but was less than 0.13 during the experiments in most cases. The pure carbon slide material readily formed a lubricating film on the contact surface, but the lubricating film was easy to break [24]. Rupture of the lubricant film would bring the friction pairs directly into contact with each other, which will cause serious friction and wear. Thus, the curve of the friction irregularly changed for the pure carbon slide material.

In Figure 6a, the average friction coefficient of the pure carbon slide composite was 0.121, whereas the friction coefficient of the C/C composite was 0.152. When the aluminum–copper alloy infiltrated the C/C composites, the friction coefficient increased from 0.152 to 0.175. The average wear rates of the C/C, C/C–Al–Cu, and pure carbon slide composites were 5.82 × 10^−7^ mm^3^/Nm, 2.56 × 10^−7^ mm^3^/Nm, and 12.32 × 10^−7^ mm^3^/Nm, respectively. During the friction processes, the pure carbon slide material could easily form a lubricating film because of the absence of the supporting role of carbon fiber and the large actual contact area. When the alloy liquid infiltrated the carbon matrix to fill the abundant defects, an important skeleton support was formed. Thus, the point-to-face contact pattern was easily obtained during the rubbing process, leading to the improvement of surface hardness, wear resistance, and friction coefficient [31].

**Figure 7:**
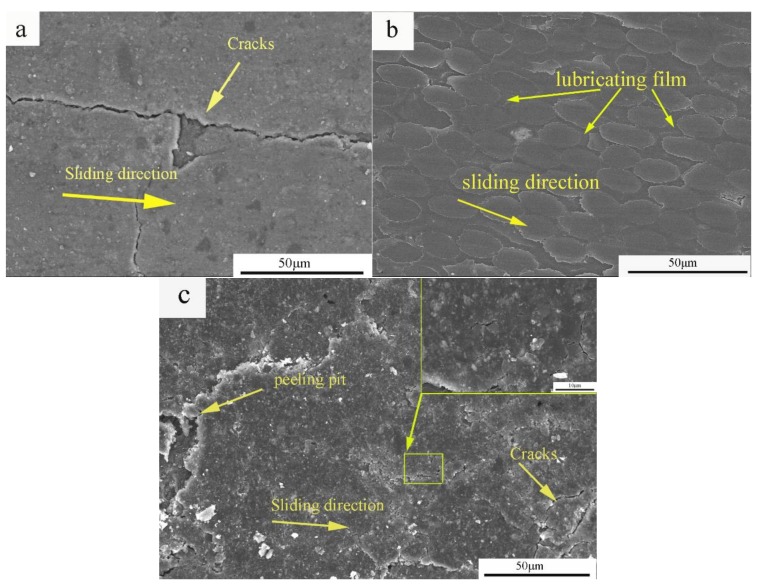
SEM micrographs of the worn surface on composites with the corresponding components. (**a**) Pure carbon slide; (**b**) C/C; and (**c**) C/C–Al–Cu.

Figure 7 shows the SEM micrographs of the worn composite surfaces. It was easier to form a lubricating film on the pure carbon slide material because of its low hardness and high microcrystalline mobility during rubbing (Figure 7a). In addition, the tribo-surface showed several microcracks caused by friction force and heat. Some of the hard particles on the surface of the carbon matrix were sheared under the action of friction force. Then, the hard particles driven by the ball cut longitudinally-plowed ditches on the contact surface of the counterface. Furthermore, hard particles accumulated on the tribo-surface. The large agglomerated particles abraded the interface, causing breakage and splitting of the lubrication, and resulted in severe adhesive wear. Thus, the wear mechanism of the composite mainly involved adhesive wear and abrasive wear.

The entire pyrolytic carbon lubricating film could be formed on the worn surface of the composite (Figure 7b). The formation of a stable transfer layer protected the mating surfaces from further direct contact, thereby resulting in a mild friction wear model.

The materials between the worn surface and microcracks were peeled off, and peeling pits were formed (Figure 7c). Moreover, mechanical and thermal stresses were continuous between the contact asperities under constant load, showing localized fatigue phenomena related to the displacement of areas. The cyclic fatigue phenomena induced the generation of peeling pits in the material. When the alloy infiltrated the C/C composites, the lubricating film contained metal particles. Some metal particles increased the hardness of the lubricating film, and other metal particles fractured or were loosened from the matrix. Accordingly, abundant large wear debris was scattered on the worn surface, contributing to severe abrasive wear [19,24]. Furthermore, several peeling pits and cracks were observed on the worn surface, suggesting an adhesive wear mechanism. 

### 3.4. Electrical Performance

**Figure 8:**
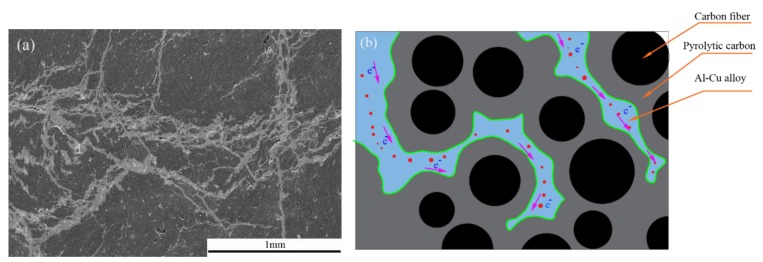
SEM micrographs of C/C–Al–Cu: (**a**) Conductive phase; and (**b**) conductive model.

In this article, the C/C composites were impregnated with metallic elements to improve electrical conductivity. The resistivity of the pure carbon slide material was 29.5 μΩm, and those of the C/C–Al–Cu (ρ = 2.2 g/cm^3^) and C/C (ρ = 1.78 g/cm^3^) composites were 1.94 μΩm and 3.56 μΩm, respectively. Results showed that the resistivity of the composites decreased, whereas the electrical conductivity increased significantly. Figure 8a,b show the microstructure of C/C after the Al–Cu alloy infiltration. Gaps were filled, and the structure became relatively dense. The aluminum–copper alloy apparently formed “network conduction” in the matrix, thus markedly improving the channel of free electron transport. The conduction mechanism was applied to explain the conductivity of the composite materials, and the well-known rule was the macroscopic seepage theory, sometimes called the percolation theory. A prominent scholar and his group, Wessling et al., studied the formation of the conductive matrix and established a dynamic interface model [31].

**Figure 9:**
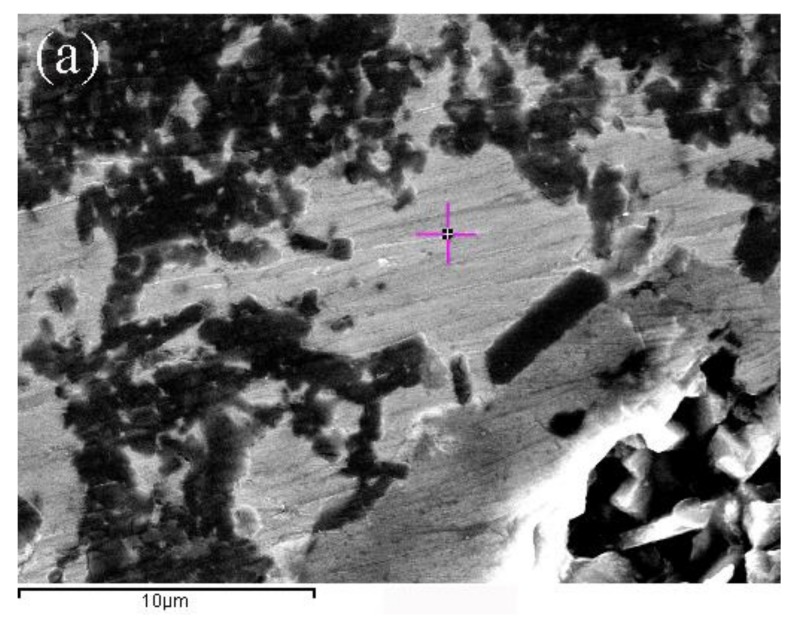

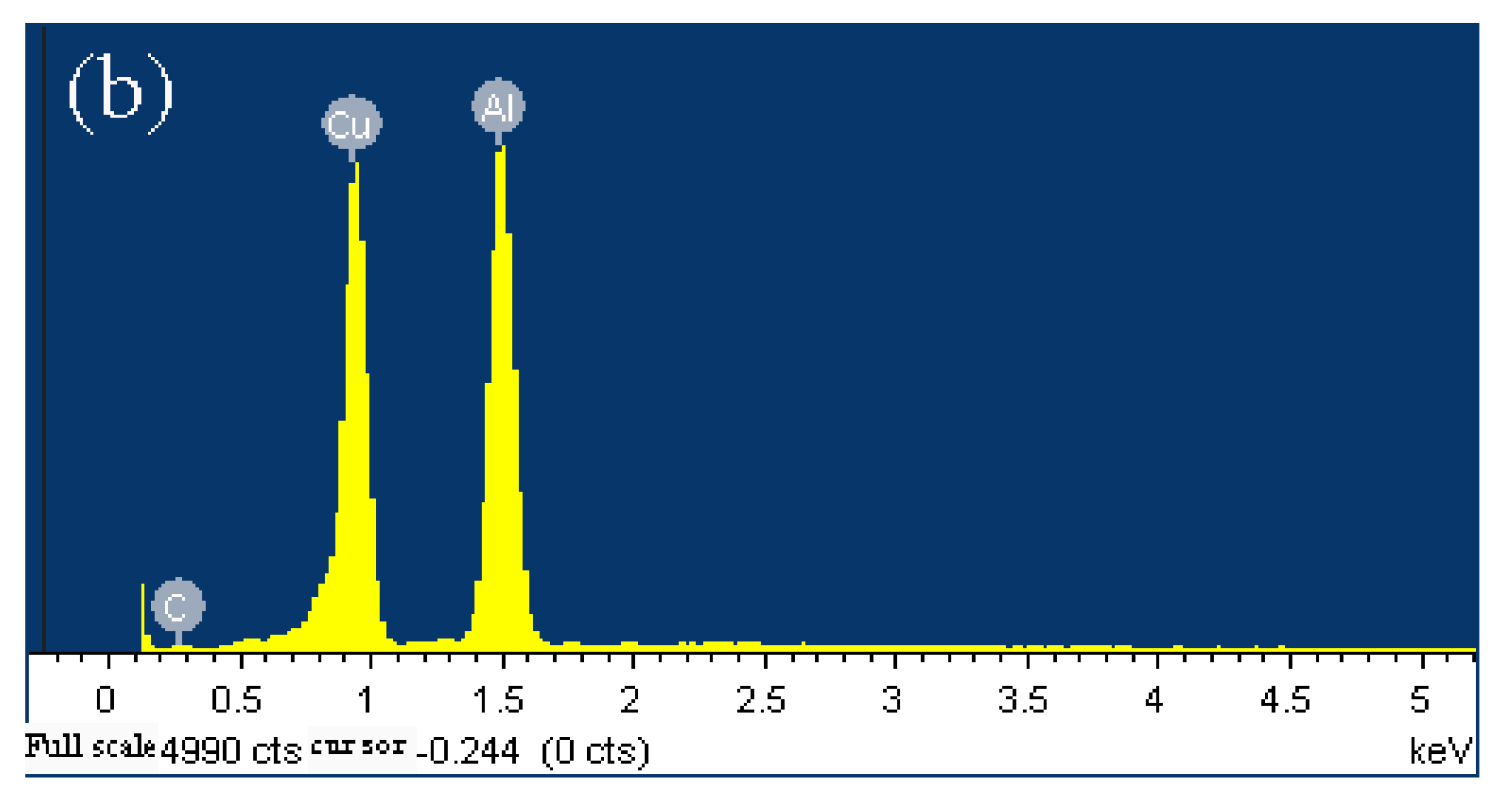
Energy spectrum pattern of infiltration alloy: (**a**) SEM micrograph of C/C–Al–Cu; and (**b**) component analysis.

Normally, molten Cu and the carbon matrix are not easily wettable at high temperature and under vacuum. Nevertheless, as seen from the EDS scanning analysis, molten copper penetrated well into the matrix because of the existence of Al_4_C_3_ (Figure 9a,b and Table 3). In summary, the conductive phase of the composite material consisted of two parts, namely, a matrix for forming material with the overall conductivity of the carbon-based conductive phase, and the network-like distribution of the metal conductive phase.

### 3.5. Wetting Performance

**Figure 10:**
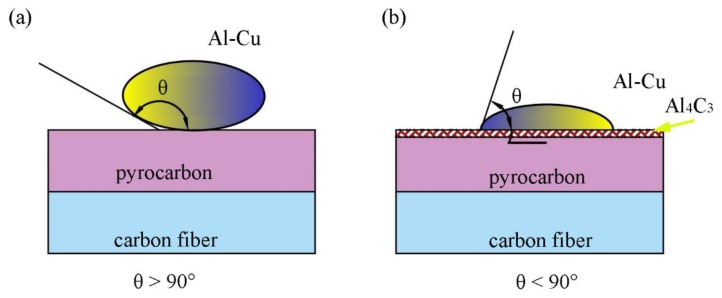

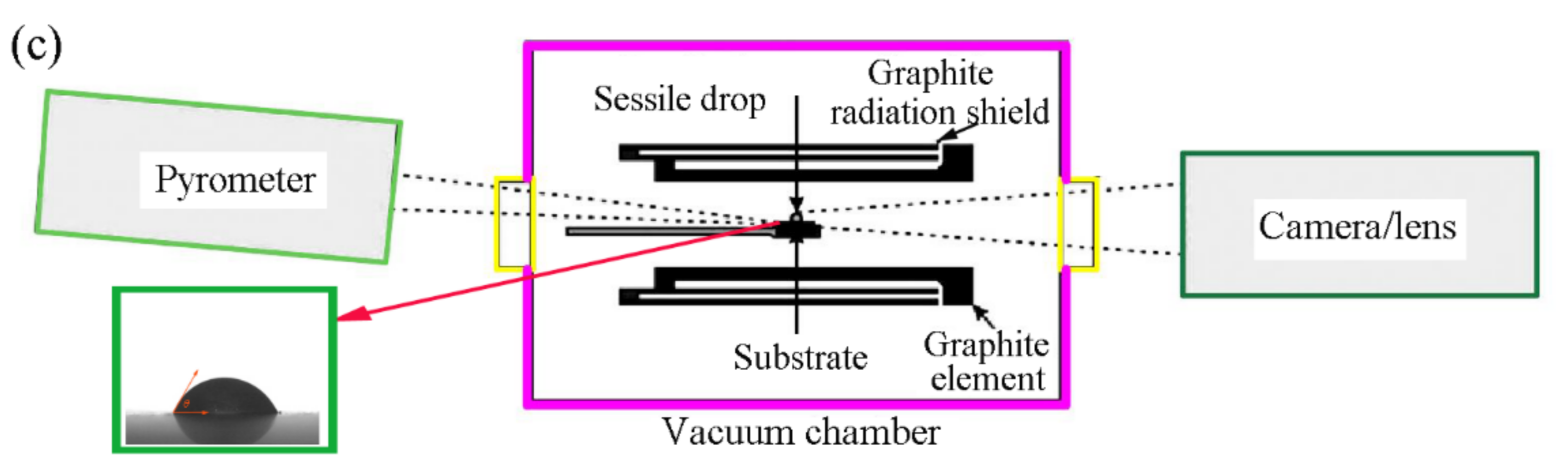
The relationship between the infiltration angles of the models and horizontal graphite heater for measuring the wetting angle (θ). (**a**) Al–Cu–Pyrocarbon; (**b**) Al–Cu–Al_4_C_3_–Pyrocarbon; and (**c**) the schematic diagram of sessile furnace.

In the Al–Cu–Al_4_C_3_–pyrocarbon system, the final contact angle on the reaction production Al_4_C_3_ was much lower than the initial contact angle of the graphite substrate, as shown in Figure 10a,b. The experimental apparatus is schematically shown in Figure 10c. The apparatus essentially consists of a horizontal graphite heater surrounded by a graphite radiation shield, located in a water-cooled vacuum chamber, with the chamber fitted with windows to allow a digital video camera (Sony XCD-SX910CR, Tokyo, Japan) to record the shape of the droplet. The contact angles and linear dimensions of the images were measured directly from the image of the drop using Video Drop Shape Analysis software [32]. Finally, the symmetry of the drop was assumed. The experimental process was as follows. The wetting experiment was carried out on the surface of the graphite substrate with an Al–Cu alloy rod. Then, the Al–Cu alloy rod with a diameter of 2 mm was cut into small pieces of around 2 mm in length and polished with No. 800 sandpaper and cleaned with ethanol to prevent further oxidation. Furthermore, the sample metal rods were placed in a vacuum chamber. When the wetting furnace attained a high vacuum of 10^−8^ bar, the sample was quickly heated to 950 °C in about 80 s, then heated to 1200 °C at a heating rate of 50 °C/min [33]. A very important factor is that aluminum is always covered with an oxide layer. However, it has been reported that after a certain temperature, the oxide layer can be removed and the contact angle reduced [34,35]. In the final experiment, a relatively stable contact of approximately 52° (θ < 90°) was obtained.

### 3.6. Discussion

**Figure 11:**
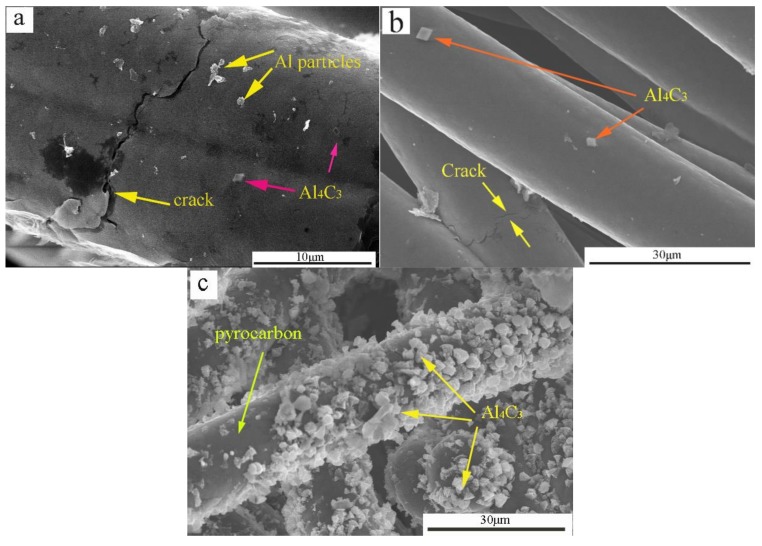
Morphology of pyrolytic carbon of carbon fibers covered with Al particle and a small quantity of Al_4_C_3_. (**a**,**b**) reacts with pyrocarbon early, and (**c**) reacts with pyrocarbon at the end.

In general, the microstructure of a material determines its macro-performance. For C/C composites, the pyrolytic carbon completely coated the carbon fibers. First, in the process of infiltration, the formation of carbide increased the bonding strength between the pyro-carbon and the Al−Cu alloy. The adhesive strength between the pyrolytic carbon matrix and Al–Cu alloy increased when the pyrolytic carbon was coated with Al_4_C_3_ particles (Figure 11a,b). Second, the Al–pyrolytic carbon systems produced Al_4_C_3_ at the end, and the interface reaction product promoted wetting (Figure 11c). Finally, the C/C–Al–Cu composites showed excellent mechanical properties when compared with the pure C/C composites.

However, Al_4_C_3_ is detrimental to the dimensional stability and mechanical properties of the composites [19,36]. Under high temperature, the carbides of the composites were formed as shown by the following chemical equation (Equation (6)):

(6) $$4{\mathrm{Al}}_{\left(l\right)}+3C_{\left(S\right)}={\mathrm{Al}}_{4}C_{3}$$

For the C/C–Al–Cu composites with carbides at the interface, the formation of carbides consumed the elemental C of the carbon fibers, leading to the transformation of interfacial bonding from mechanical to chemical bonding. The wetting behavior mechanism should be analyzed. These results were consisted with the previous reports [37,38,39]. In Equations (7) and (8), θ is the wetting angle, and the equilibrium value of the contact angle θ, which is used to define the wetting behavior of the liquid, obeys the classical Young equation [40]. In this article, the Al–pyrolytic carbon systems produced a small quantity of Al_4_C_3_ at the end, and the interface reaction product decreased the wetting angle (θ < 90°) [41,42].

(7) $$\delta_{g\cdot s}=\delta_{l\cdot s}+\delta_{g\cdot l}\cdot \cos\theta$$

(8) $$\cos\theta =\frac{\delta_{g\cdot s}-\delta_{l\cdot s}}{\delta_{g\cdot l}}$$

where δ is the surface tension. The subscripts s, g, and l refer to solid, gaseous, and liquid. When δ_g∙s_ > δ_l∙s_, cos θ > 0, and θ < 90°, the alloy can wet the graphite material; in contrast, the alloy cannot wet the graphite material when δ_g∙s_ < δ_l∙s_, cos θ < 0, and θ > 90°.

During the friction process, the entire graphite lubricating film was formed on the worn surface of the C/C–Al–Cu composites with metal particles (Figure 7c). The formation of a stable transfer layer protected the mating surfaces from further direct contact, leading to mild friction and wear [23] as the metal particles in the carbon matrix protected the softer matrix during abrasive sliding and strengthened the matrix. Nevertheless, the difference should not be ignored. The C/C composite lacked hard particles in the matrix. Thus, the graphite lubricating film of the C/C composite was easily broken during the friction process, thus explaining the increase in the friction coefficient of C/C–Al–Cu. 

As seen in Figure 8a, the continuous and uniform electrical network of the Al–Cu phase formed in the composite, and the electrical resistivity of the composite was lower than that of the strips for current collectors currently used in railways (29.5 μΩm, CY280). Hence, a continuous electric network of the Al–Cu phase can form, thereby explaining the decrease in the electrical resistivity value when compared with that of the pure C/C composites.

**Figure 12:**
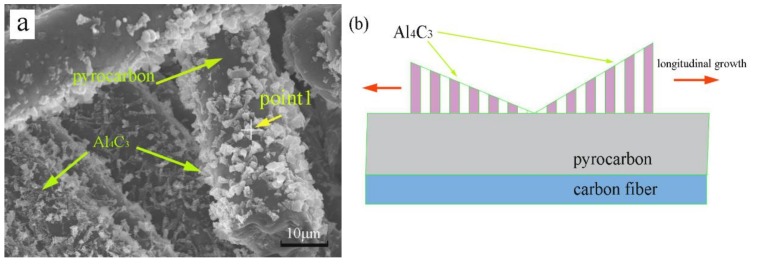

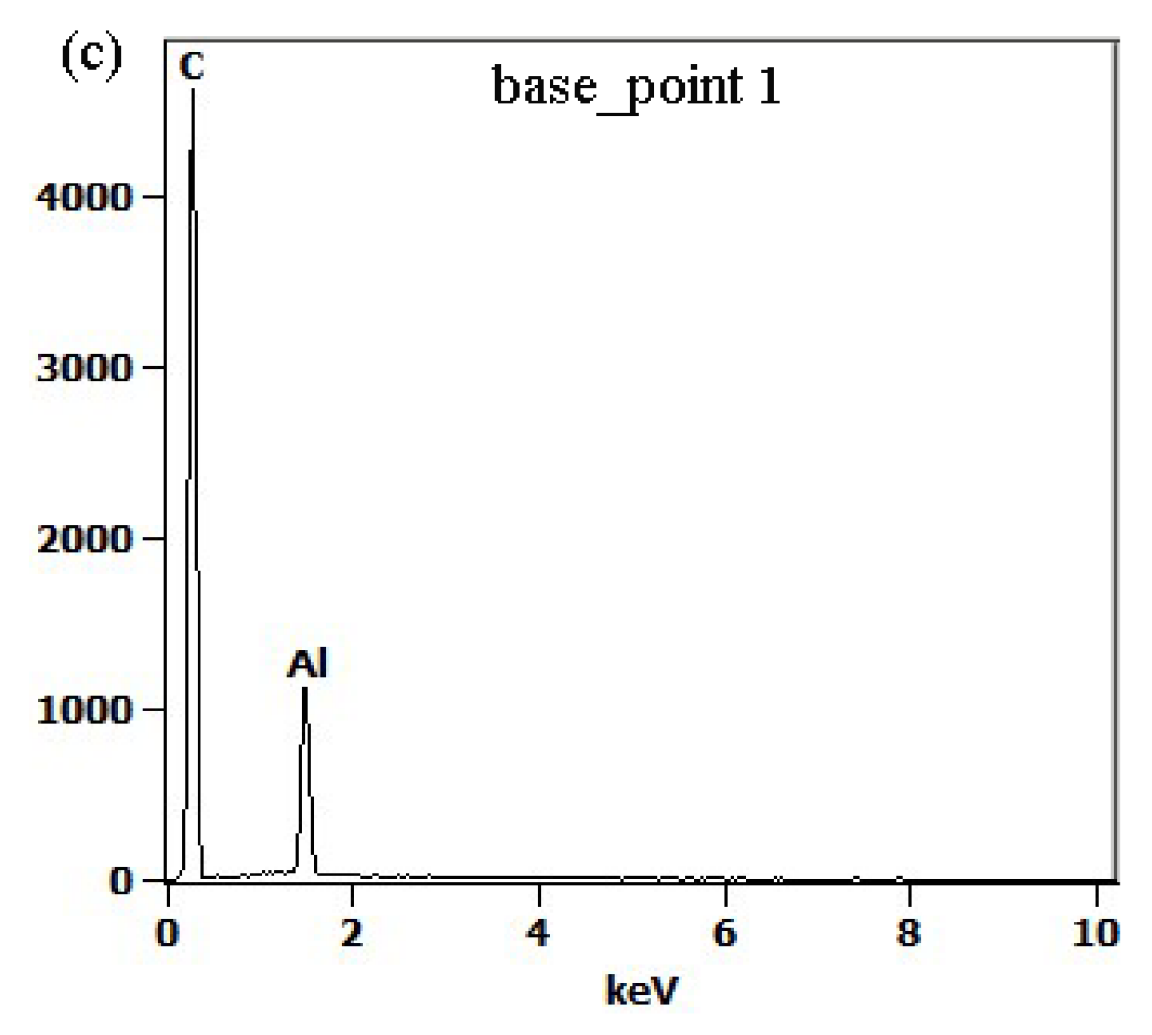
The Al_4_C_3_ particles formed on the surface of pyrocarbon: (**a**,**c**) SEM and EDS patterns of the particles; and (**b**) the formation mechanism of Al_4_C_3_ particles.

The EDS patterns of the particles that were considered as Al_4_C_3_ are shown in Figure 12a,c. From the results of the SEM, EDS, and XRD observations, the formation of Al_4_C_3_ could be divided into two stages: Nucleation and growth. For the nucleation stage, the carbon atoms diffused quickly during the fabrication process and Al_4_C_3_ nucleared at defects of the pyrocarbon surface as shown in Figure 12a,c. For the growth stage, the carbon atoms diffused to both the matrix and the surface of Al_4_C_3_. Therefore, Al_4_C_3_ grew with the sedimentary carbon atom. During the growth process, Al_4_C_3_ formed a lamellar morphology on the pyrocarbon surface as shown in Figure 12b. In the end, the main growth direction of Al_4_C_3_ was longitudinal [43].

## 4. Conclusions

C/C–Al–Cu composites were prepared by infiltrating the alloy under vacuum and at high temperature. Mechanical and tribological properties and electrical performance were investigated. The experimental results led to the following conclusions:

A simple and effective strategy was developed to prepare C/C–Al–Cu composites. SEM, ED, and XRD showed that the Al_4_C_3_ particles formed on the surface of the pyrocarbon matrix by strongly affecting the interface strength. Mechanical tests verified that the bending property of C/C–Al–Cu and the pure carbon slide material composites were 189 MPa and 85 Mpa, respectively. Furthermore, the compressive strength of C/C–Al–Cu and the pure carbon slide material were 213 MPa and 102 MPa, respectively. A continuous electric network of the Al–Cu phase could be formed, and this possibility may explain the decrease in electrical resistivity value. This unique microstructure was beneficial for electrical properties. The resistivity of the C/C, C/C–Al–Cu, and pure carbon slide composites were 3.56 μΩm, 1.94 μΩm, and 29.5 μΩm, respectively. For the friction properties, the friction coefficients of the C/C, C/C–Al–Cu, and pure carbon slide composites were 0.152, 0.175, and 0.121, respectively. Furthermore, the wear rate of the C/C–Al–Cu composites reached a minimum value of 2.56 × 10^−7^ mm^3^/Nm during the friction process. Therefore, the C/C–Al–Cu composites had moderate-intensity adhesive wear in comparison to the C/C composite, which adequately explained the slight increase in friction coefficient value. Finally, the Al–pyrolytic carbon systems produced a small quantity of Al_4_C_3_ during the process of infiltration, and the interface reaction product decreased the wetting angle (θ). Consequently, a relatively stable contact angle of approximately 52° (θ < 90°) was obtained.

## Figures and Tables

**Table 1:** Major properties of the industry standard.

Type	Density (g/cm^3^)	Resistivity (μΩm)	Friction Coefficient	Wear Rate (mm^3^/Nm)	Mechanical Properties (MPa)	
					Bending Strength	Compressive Strength
Reference standard	≤3.0	≤12	≤0.2	≤1.0 × 10^−6^	≥80	≥50

**Table 2:** Mechanical properties of different pantograph sliders (MPa).

Type	Industry Standard	(Copper/Graphite) Powder Metallurgy	Sintered Alloy	Pure Carbon Slide (CY280)	C/C Composites (ρ = 1.3 g/cm^3^)	C/C–Al–Cu Composites (ρ = 2.24 g/cm^3^)
Bending strength	≥80	≥80	≥220	85	66	189
Compressive strength	≥50	≥56	300	102	74	213

**Table 3:** Quantitative analysis of the alloy energy spectrum.

Element	wt %	at %
C K	4.49	13.73
Al K	39.62	53.95
Cu L	55.89	32.31
Total weight	100.00	
